# The impact of neuronal Notch-1/JNK pathway on intracerebral hemorrhage-induced neuronal injury of rat model

**DOI:** 10.18632/oncotarget.12094

**Published:** 2016-09-17

**Authors:** Maohua Chen, Jun Sun, Chuan Lu, Xiandong Chen, Huajun Ba, Qun Lin, Jianyong Cai, Junxia Dai

**Affiliations:** ^1^ Department of Neurosurgery, Wenzhou Central Hospital, Affiliated Dingli Clinical Institute of Wenzhou Medical University, Wenzhou, China

**Keywords:** Notch-1, JNK/c-Jun, ICH, Rat

## Abstract

Notch signaling is a highly conserved pathway that regulates cell fate decisions during embryonic development. Notch activation endangers neurons by modulating NF-κB and HIF-1α pathways, however, the role of Notch signaling in activating JNK/c-Jun following intracerebral hemorrhage (ICH) has not been investigated. In this study, we used rat ICH models and thrombin-induced cell models to investigate the potential role of Notch-1/JNK signals. Our findings revealed that Notch-1 and JNK increased in hematoma-surrounding neurons tissues following ICH during ischemic conditions (all p<0.05). Besides, the expression of active caspase-3 protein was also up-regulated after ICH. According to *in-vitro* assays, the expression of Notch-1, p-JNK, and active caspase-3 were all up-regulated in cell viability-decreasing ICH cell models (all p<0.05). However, blocking of either Notch-1 or JNK suppressed the phosphorylation of JNK and the expression of active caspase-3, and cell viability was obviously ameliorated. In conclusion, this work suggested Notch-1 activates JNK pathway to induce the active caspase-3, leading to neuronal injury when intracerebral hemorrhage or ischemia occurred. Thus the Notch-1/JNK signal pathway has an important role in ICH process, and may be a therapeutic target to prevent brain injury.

## INTRODUCTION

Intracerebral hemorrhage (ICH) is a serious cerebrovascular condition leading to high mortality and morbidity in adults. The global incidence of ICH is increasing year by year, with a trend towards growing incidence at a younger age [1]. Despite significant progress in clinical treatment, the 5-year mortality rate remains over 50% (52% for males, 56% for females) in ICH patients older than 45 years [2]. Even after surgical treatment, 20% of ICH patients experience varying degrees of neurological dysfunction, requiring long-term hospitalization and rehabilitation [3–4]. Thus, ICH not only causes serious morbidity and mortality in patients, but also incurs a serious burden on families and society. The pathological mechanisms of hematoma after ICH within brain parenchyma trigger a series of adverse events and severe neurological deficits. In recent years, progress has been made in ICH research. However, the effects of treatment options on ICH were not satisfying [5]. Thus, it is essential to understand the potential molecular mechanisms of ICH-induced brain injury.

During early neural development, Notch is highly expressed in the neural precursor cells and plays a critical role in regulating neural proliferation and differentiation [6]. Besides its critical functions in a variety of cell-fate decisions during development [6, 7], The Notch pathway is an important signal for cell differentiation and cell survival [8, 9], and is known to play critical roles in kidney development [10]. Notch proteins, including Notch1-Notch4, are transmembrane receptors and bind many ligands including Delta-like (Dll) 1, Dll3, Dll4, Jagged1 and Jagged2 [11]. The activation of Notch results in the sequential proteolytic cleavage of the Notch receptor, which releases Notch intracellular domain (NICD) into the nucleus, which in turn activates downstream gene transcription including that of the Hes and Hey genes [12, 13]. Nowadays, there is increasing evidence that the Notch signaling pathway is extensively involved in HG-induced podocyte apoptosis [14, 15]. Therefore, targeting the Notch pathway may provide novel insights into the mechanism underlying diabetic nephropathy and possible targets for drug development. Because the role of Notch-1-JNK in the ICH is still raveled, here we investigated the role of Notch-1-JNK in ICH-induced neuronal injury.

In the present study, we used rat ICH models and thrombin-induced cell models to investigate the potential role of Notch-1-JNK signals. Our findings revealed that JNK could be phosphorylated by Notch-1 to induce the active caspase-3 and neuronal injury when intracerebral hemorrhage or ischemia occurred. Thus the Notch-1/JNK signal pathway has an important role in ICH process, and may be a therapeutic target to prevent brain injury.

## RESULTS

### Expression status of Notch-1, JNK, and active caspase-3 in samples surrounding ICH hematoma

**Figure 1:**
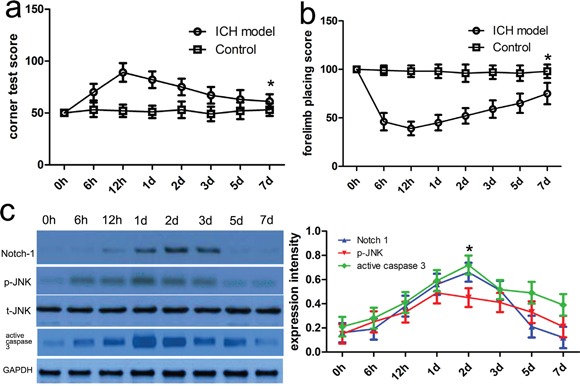
Notch-1 protein level was increased adjacent to the hematoma after ICH Neurofunctional assessments were carried out in rats with or without ICH. Forelimb placing test **a.** and corner turn test **b.** scores at different survival time points following ICH. **c.** Western blot analysis was conducted to investigate the expression profile of relevant proteins in the ipsilateral basal ganglia around the hematoma at different time points after ICH. Quantification analysis was defined as the relative density of relevant proteins to GAPDH at each time point. GAPDH was used as a loading control. Results shown are the mean ± SEM of repeated independent experiments. *p<0.001, compared with control, one-way ANOVA.

To characterize the expression profile and activity of relevant proteins in tissues surrounding ICH hematoma, we designed a rat ICH model and selected the rat model with defected neurological functions using neurofunctional assessments as mentioned above (Figure 1a-1b). In this present study, western blot was carried out to investigate the temporal expression patterns of relevant proteins including Notch-1, JNK, and active caspase-3 in tissues adjacent to the hematoma. Compared with the control group, we found that Notch-1 expression began to increase after post-treatment 12 hour following ICH and was peaked at 2 days (p<0.05) (Figure 1c). Consistent with the Notch-1 expression, p-JNK or active caspase-3 protein expression was also confirmed by western blot. The expression of p-JNK and active caspase-3 protein was enhanced after post-treatment 6 hour following ICH and was peaked at 48 hours (p<0.05) (Figure 1c). It should be noted that t-JNK expression was not obviously altered in comparison with that in different times.

### Expression of NICD in samples surrounding ICH hematoma is enhanced

**Figure 2:**
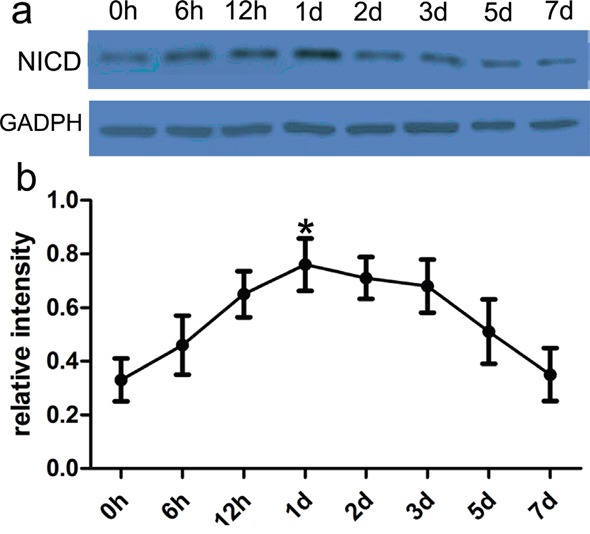
Notch-1 activity was highly altered in rat ICH model In the present study, western blot analysis of Notch-1 activity in ipsilateral basal ganglia adjacent to the hematoma was performed at different time points following ICH. **a.** The immune complex was subjected to immunoblot analysis with NICD antibody. GAPDH was used as a loading control. **b.** Quantification analysis showed the increased Notch-1 activity after ICH. Results shown are the mean ± SEM of repeated independent experiments. *p<0.01, compared with control, one-way ANOVA.

To figure out Notch-1 activity after ICH, we conducted the immunoblotting to detect the expression of NICD. Here, NICD protein was extracted from the control and ICH group, and then NICD was further analyzed to appraise Notch-1 activity. In the present study, the expression of NICD was very low in the control group. However its expression was increased at postoperative 2 hours and peaked at 2 days (Figure 2). These findings revealed that Notch-1 protein activities were highly elevated, and relevant to ICH-induced neuronal biology.

### Notch-1 induces JNK signals to meditate neuronal apoptosis

**Figure 3:**
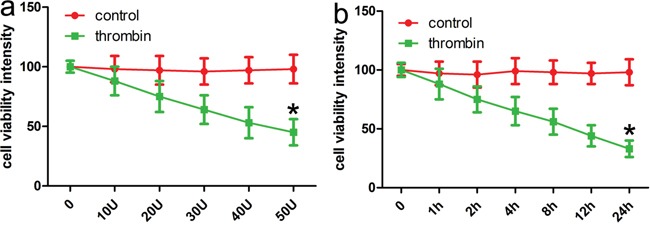
CCK-8 assays identifies that thrombin induces ICH cell models **a.** Thrombin (40U/ml) reduced neuronal viability in a dose-dependent manner at post-treatment 24 h. **b.** Thrombin (40U/ml) reduced neuronal viability in a time-dependent manner. Results shown are the mean ± SEM of repeated independent experiments. *p<0.001, vs control; one-way ANOVA was used.

**Figure 4:**
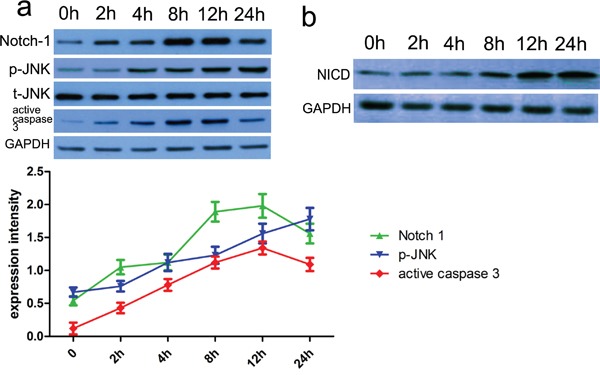
The relevant proteins and Notch-1 activity were altered in thrombin-induced cell model **a.** Cells were pre-treated with thrombin (40U/ml). After that, cells lysates were subjected to immunoblotting. Subsequently, Notch-1 pathway-related proteins including p35/p25, Notch-1, p-JNK and active caspase 3 were assessed. Protein expression level was calculated using Image J Pro software. GAPDH was used as a normalization control. Each bar represents the mean ± SEM of three independent experiments; *p<0.001, compared with control (0 h), using one-way ANOVA with the LSD post hoc test. **b.** We used anti-Notch-1 antibody to immunoprecipitate Notch-1, and then assessed its activity with DLL4 as a substrate. The immune complex was subjected to immunoblot analysis with Notch-1 antibody to conform the efficiency of immunoprecipitation. GAPDH was used as a loading control. Quantification analysis showed the increased Notch-1 kinase activity after ICH. Results shown are the mean ± SEM of repeated independent experiments. *p<0.001, compared with control, one-way ANOVA.

**Figure 5:**
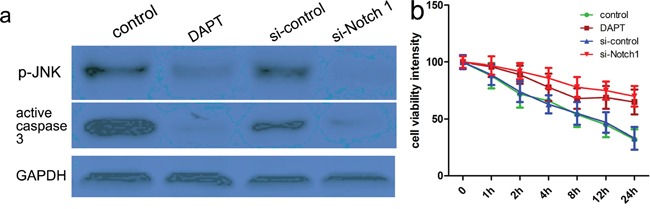
Notch-1 induces JNK signals to meditate neuronal injury Cells were pretreated with thrombin (40U/ml), and then DAPT was added 24 h before. At the same time, cells were also transfected with si-control or si-Notch-1 for 48 hours. After that, cells lysates were subjected to western blotting **a.** Protein expression level was calculated using Image J Pro software. GAPDH was used as a normalization control. Each bar represents the mean ± SEM of three independent experiments; *p<0.001, compared with control. one-way ANOVA with the LSD post hoc test. **b.** CCK-8 assays identified that DAPT or si-Notch-1 could attenuate thrombin-induced neuronal apoptosis. Results shown are the mean ± SEM of repeated independent experiments. *p<0.001, compared with control, one-way ANOVA.

To determine a role of Notch-1 in the phosphorylation of JNK, blocking Notch signaling activation by a γ-secretase inhibitor, DAPT was applied, and then relevant proteins including p-JNK and active caspase 3 were detected in the present study. Firstly, the CCK-8 assay showed thrombin successfully induced low cell viability in a dose and time dependent manner (Figure 3a, 3b). Consistent with rat ICH model, relevant proteins also showed altered expression with induction of thrombin (Figure 4). Subsequently, DAPT markedly blocked the expression of p-JNK and active caspase 3 in the thrombin-induced cell model (Figure 5a), at the same time, the expression of NICD was also inhibited in comparison with control. Besides, we knocked down the endogenous Notch-1 with Notch-1 siRNAs, and found knockdown of Notch-1 robustly blocked the elevated level of p-JNK and active caspase 3 in the thrombin-induced cells (Figure 5a), which was consistent with the DAPT treatment. According to flow cytometry, DAPT obviously attenuated the decreased neuronal cell growth induced by thrombin. Besides, the Notch-1 siRNA also attenuated the thrombin-inhibited cell survival (Figure 5b). These results indicated that Notch-1 directly or indirectly stimulated the phosphorylation of JNK to contribute to neuronal injury.

### JNK inhibitor bentamapimod affects the transduction of Notch-1 signals

**Figure 6:**
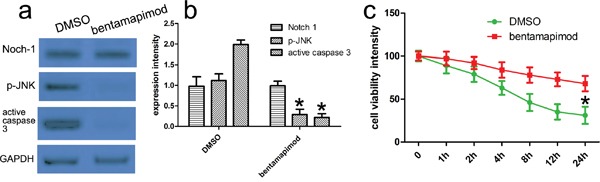
JNK inhibitor bentamapimod affects the transduction of Notch-1 signals **a.** Cells were treated as described above. JNK inhibitor bentamapimod was added in corresponding group. Expression of relevant proteins including phosphorylated JNK, total JNK and active caspase 3 proteins was detected with western blot analysis. GAPDH was used as a loading control. **b.** Quantitative analysis was performed with Image J. *p<0.001, compared with control. one-way ANOVA with the LSD post hoc test. **c.** CCK-8 assays identifies that bentamapimod could attenuate thrombin-induced neuronal injury. Results shown are the mean ± SEM of repeated independent experiments. *p<0.001, compared with control, one-way ANOVA.

To further determine the signals of Notch-1-JNK in the activation of JNK, we used the JNK specific inhibitor bentamapimod to observe its effects on cell viability. We carried out western blot to analyse the expression of relevant proteins including Notch-1, phosphorylated and total JNK protein, and active caspase 3. Our results revealed the bentamapimod inhibited the phosphorylation of JNK and the expression of active caspase-3 protein, while bentamapimod did not affect the expression of Notch-1 (Figure 6a, 6b). To further determine the effect of JNK on neuronal viability, we conducted the CCK8 assay, and found bentamapimod could attenuate thrombin-induced neuronal injury (Figure 6c). These findings indicated that Notch-1 regulated the phosphorylation of JNK, and the phosphorylated JNK was involved in neuronal apoptosis.

### The silencing of JNK attenuates Notch-1 induced neuronal injury

**Figure 7:**
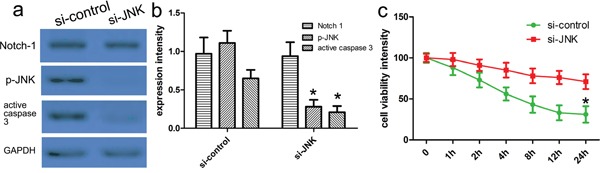
Notch-1-JNK pathway regulated neuronal apoptosis and JNK phosphorylation Western blot showed siRNA-silenced JNK in primary cortical neurons with thrombin treatment **a.** GAPDH was used as a loading control. **b.** Quantitative analysis was performed with Image J. *p<0.001, compared with control. one-way ANOVA with the LSD post hoc test. **c.** CCK-8 assays identifies that knockdown of JNK could attenuate thrombin-induced neuronal apoptosis. Results shown are the mean ± SEM of repeated independent experiments. *p<0.001, compared with control, one-way ANOVA.

To elucidate the significance of JNK in Notch-1-JNK-induced neuronal injury when ICH occurred, we used specific JNK siRNAs to block the expression of JNK, and then observed Notch-1 activity and the relevant proteins in thrombin-induced neuronal cell model. Western blot analysis revealed that the knockdown of JNK affected Notch-1 pathway in thrombin-induced neuronal cell models compared with control siRNA. Subsequently, downregulated p-JNK activity also repressed the expression of active caspase-3 (Figure 7a, 7b). Based on the CCK-8 assay, the knockdown of JNK can reverse reduction of neuron cell viability caused by thrombin (Figure 7c). These findings indicated Notch-1-JNK pathway could contribute to thrombin-induced neuronal injury.

## DISCUSSION

In recent years, the disrupted Notch-1 expression is associated with some neurodegenerative or cardiovascular diseases [18, 19]. During early neural development, Notch is highly expressed in the neural precursor cells and plays a critical role in regulating neural proliferation and differentiation [6]. Besides its critical functions in a variety of cell-fate decisions during development [6, 7], The Notch pathway is an important signal for cell differentiation and cell survival [8, 9], and is known to play critical roles in kidney development [10]. In addition, the c-Jun N-terminal kinases (JNKs), as members of the mitogen-activated protein kinase (MAPK) family, mediate eukaryotic cell responses to a wide range of abiotic and biotic stress insults. JNKs also regulate important physiological processes, including neuronal functions, immunological actions, and embryonic development, via their impact on gene expression, cytoskeletal protein dynamics, and cell death/survival pathways [20–22]. Although the JNK pathway has been under study for >20 years, the potential Notch-1-JNK pathway in neuronal injury following ICH is always unclear.

In the present study, we used a rat ICH model to simulate the pathological processes of ICH. Meanwhile we also applied a thrombin-induced neuronal cell model. With the help of these models, we found that the expression of Notch-1, p-JNK and active caspase-3 began to increase in the hematoma-surrounding tissues after post-treatment 6 hour following ICH and was peaked at 48 hours. At the same time, Notch-1 and NeuN were co-expressed in neurons of hematoma-surrounding tissues. These results indicated Notch-1 specifically actions in the neurons. It should be noted that highly activated Notch-1 is associated with many neurodegenerative disorders such as Alzheimer's or Parkinson's diseases. Notch-1 may be an important link between disease-initiating factors and cell death effectors. In this work, we identified that Notch-1 protein activities were highly elevated, and relevant to ICH-induced neuronal biology. Abnormal Notch-1 signaling induces dysfunction of individual proteins and has an essential role in interactions of proteins in different neurodegenerative diseases. Thus the downstream effectors of Notch-1 needed to be investigated in our study.

Next, we used thrombin-induced cell models and *in-vitro* assays to identify the potential Notch-1-JNK pathway. It is reported that DAPT is widely used as a biological tool in cell cycle, cancer, apoptosis and neurobiology studies, which is currently recommended as a useful drug to treat cancers, neurodegenerative diseases, inflammation, etc. In our study, DAPT markedly blocked the expression of p-JNK and active caspase 3, and inhibited the kinase activity of Notch-1 in the thrombin-induced cell model. Accordingly knockdown of Notch-1 also robustly blocked the Notch-1-JNK pathway. Furthermore, the block of JNK affected the phosphorylation of Notch-1-induced JNK. These results suggested that Notch-1-JNK signals play a substantial role in ICH progression, including cell injury.

Functional analysis showed that the blocking of Notch-1 or JNK can improve neuronal cell viability, indicating interruption of the Notch-1-JNK pathway attenuates DNA-damage-induced neuronal cell cycle re-entry and can be utilized as a useful target. Mechanically, Notch-1 pathways involve some common signal pathways, including the extracellular signal-regulated kinase 1/2, and c-jun N-terminal kinase 3 [23–25]. Consistent with our studies, these findings further demonstrate the role of JNK in DNA damage-induced neuronal death.

In conclusion, our data identified that JNK could be phosphorylated by Notch-1 to induce the active caspase-3 and neuronal injury when intracerebral hemorrhage or ischemia occurred. Thus the Notch-1-JNKsignal pathway has an important role in ICH process, and may be a therapeutic target to prevent brain injury.

## MATERIALS AND METHODS

### Animal procedures

The protocol was approved by the Medical Ethics Committee of Chongqing Medical University and Wenzhou Medical University. Animal experiments were performed according to the guidelines for the care and use of animals as established by Wenzhou Medical University. A total of 270 adult male Sprague-Dawley rats (age, 8–10 weeks; weight, 180–220 g) were obtained from the Animal Center of Chongqing Medical University and Wenzhou Medical University. Rats were housed at 25°C under a 12-h light/dark cycle, with ad libitum access to food and water. Animal care and surgical procedures were carried out in accordance with the Guide for the Care and Use of Laboratory Animals promulgated by the National Research Council in 1996. All efforts were made to minimize the suffering and number of animals used in experiments.

### Autologous blood-induced ICH model preparation

Briefly, the rats were anesthetized by intraperitoneal injection with 10% chloral hydrate (400 mg/kg; from the Pharmacy of Wenzhou Medical University) and fixed in a prone position on a stereotactic frame (Stoelting Co., Wood Dale, IL, USA). Following a scalp incision, a small cranial burr was drilled near the right coronal suture 3.2 mm lateral to the midline. Autologous blood sample (50 μL) was slowly injected into the right globus pallidus (1.4 mm posterior and 3.2 mm lateral to the bregma and 5.6 mm ventral to the cortical surface) with a 5-μl Hamilton syringe (The Gaoge Company, Shanghai, China) over 5 min, with the needle left in place for a further 5 min. The bone hole was sealed with bone wax, and the wound was sutured. Each animal was placed in a warm box to recover individually. Sham-operated rats were administered an equal volume of saline alone. Rectal temperatures were monitored during all operations and maintained at 37.5°C with a feedback controlled heating pad (Huaibei Zhenghua Biology Instrument Equipment Co., Ltd., Huaibei, China).

### Forelimb placing test

The ability of a mouse to respond to a vibrissae-elicited excitation by forward moving of its forelimb was evaluated with the forelimb placing test, as previously described [16]. Briefly, animals held by their trunk, were positioned parallel to a table top and slowly moved up and down, allowing the vibrissae on one side of the head to brush along the table surface. Refractory placements of the impaired (left) forelimb were evaluated and a score was calculated as number of successful forelimb placements out of 10 consecutive trials.

### Corner turn test

Corner turn test was also conducted at 24 and 72 hours after ICH to evaluate the neurological deficits as previous [17]. Briefly, rats were allowed to walk into a 30-degree corner. When exited the corner, the rat could turn either to the left or the right, and this choice was recorded. Trials were repeated 10 times with 30 seconds interval, and the percentage of right turns was calculated.

### Antibodies and regents

The antibodies used in this study were as follows: monoclonal anti-active caspase-3 antibodies were obtained from Cell Signaling Technology; anti-NICD, p-JNK, t-JNK, and GAPDH were obtained from Abcam. Anti-Notch-1 was obtained from Santa Cruz Biotechnology. JNK-specific inhibitor bentamapimod (CAS 848344-36-5) were purchased from Santa Cruz Biotechnology. The regents used in this study were as follows: Dulbecco's Modified Eagle Medium (DMEM), penicillin/streptomycin, Trypsin-EDTA, and phosphatases inhibitor cocktails were purchased from Sigma-Aldrich; NEUROBASAL™Medium, horse serum, and fetal bovine serum (FBS) were purchased from Gibco. Cell counting kit-8 (CCK-8) kits and Protease Inhibitor Cocktail Tablet were obtained from Roche. γ-secretase inhibitor DAPT (Selleck Chemicals) was administrated to block Notch signaling pathway.

### Western blot analysis

For the protein analysis, the cells were harvested at 12~24 h following different treatments, as described above, and washed with cold PBS and then incubated in ice-cold RIPA buffer. Cell lysates were sonicated for 30 s on ice and lysed at 4°C for 60 min. Then, the cell lysates were centrifuged at 12,000 g for 30 min at 4°C. Protein concentrations in the supernatants were determined by the BCA reagent. Total protein was separated by denaturing 8–12% SDS-polyacrylamide gel electrophoresis, which was resolved over and electrotransferred by semidry blotting (Bio-Rad Laboratories, Shanghai) onto a nitrocellulose membrane. The membrane was incubated with antibodies overnight at 4°C, and then with peroxidase-conjugated secondary antibody (Santa Cruz Biotech, Santa Cruz, CA, 1:1000 dilution), visualized by chemiluminescence (GE, Fairfield, CT, USA).

### Primary cortical neuronal cultures

According to previous reports, isolated neuronal cells were seeded into plates pre-coated with 100 mg/L poly-L-lysine at a density of 2×106 cells/well in 6-well plates or 2×104 cells/well in 96-well. After being incubated at 37 °C with 5 % CO2 for 4 h, the medium was replaced with NEUROBASAL™ Medium supplemented with 2% B27, 0.5 mM glutamine, and 1 % antibiotic-antimycotic. The isolated cells were used for experiments after 7 days of culture. To study apoptosis experiment, cells were incubated in NEUROBASAL™ medium without supplement for 24 h prior to treatment with thrombin at various time points.

### Cell viability assay

Cells were seeded onto 96-well plates at 3 × 103 cells/well. The medium was replaced with the corresponding serum-free medium for 24 h, and then serum-free medium was replaced with complete medium. Then 10 μL/well CCK-8 solutions was added and incubated with the plates for 3 h, and the absorbance was determined at 450 nm using an MRX II microplate reader (Dynex, Chantilly, VA, USA). The experiments were repeated, at least five replicates of each treatment.

### siRNA transfection

To inhibit Notch-1 and JNK expression, Notch-1 and JNK siRNA was a generous gift from Dr Zhengbu Liao (Chongqing Medical University). Transient transfection was performed using the Lipofectamine 2000 reagent (Invitrogen, California, USA) according to the manufacture's protocol. Cells were plated at a density of 5 × 105 cells in 6-well plates and were then transfected with siRNAs using Lipofectamine 2000 reagent diluted in Opti-MEM Reduced Serum Medium 24 h later. The final concentration of siRNA was 80 nM. Complete medium free of antibiotics was added to each well 6 h after transfection. Cells were trypsinized and harvested for western blot analysis at the indicated times.

### Statistical analysis

Data were expressed as mean ± SEM. Neurobehavioral data were analyzed by using Kruskal-Wallis One Way Analysis of Variance on Ranks, followed by the Student-Newan-Keuls Method. All other data were analyzed by using One Way Analysis of Variance followed by Tukey post hoc test. P value < 0.05 was considered statistically significant. All statistical analyses were performed by using SigmaPlot 10.0 for Windows (Systat Software Inc., San Jose, CA).
